# Comparison of visfatin levels before and after non-surgical periodontal therapy: A systematic review and meta-analysis

**DOI:** 10.1371/journal.pone.0315035

**Published:** 2025-02-19

**Authors:** Zahra Tajik, Hamid Mehrafarid, Mojtaba Bayani, Amir Almasi-Hashiani

**Affiliations:** 1 Student Research Committee, Arak University of Medical Sciences, Arak, Iran; 2 Department of Periodontics, School of Dentistry, Arak University of Medical Sciences, Arak, Iran; 3 Department of Epidemiology, School of Health, Arak University of Medical Sciences, Arak, Iran; 4 Traditional and Complementary Medicine Research Center, School of Medicine, Arak University of Medical Sciences, Arak, Iran; International Medical University, MALAYSIA

## Abstract

Periodontitis is an inflammatory disease and involves a severe inflammation of the periodontium. On the other hand, visfatin is known as one of the inflammatory markers and can probably preserve inflammation in immune cells. Therefore, the purpose of this systematic review and meta-analysis is to compare the mean visfatin level before and after non-surgical periodontal treatment (NSPT). In this systematic review and meta-analysis, to find relevant studies, PubMed, Web of Science and Scopus were searched. Google Scholar was used to review gray literature. Standardized mean difference (SMD) along with 95% confidence interval (95%CI) was calculated. To perform the meta-analysis, in cases where there was significant heterogeneity between the studies, the random-effects model was used, otherwise the fixed-effects model was used. Sixteen studies were included in the meta-analysis. The results show that one month after the NSPT, there was no significant difference in the mean visfatin level of GCF (SMD: -3.91, 95%CI: -9.83, 2.01, p = 0.195, I-square, 96.6%, random effect model, n = 2) and serum (SMD: -0.33, 95%CI: -0.98, 0.33, p = 0.332, fixed effect model, n = 1), but 2, 3 and 6 months after NSPT, the mean visfatin level of GCF and serum decreased significantly. There is no significant difference one month after NSPT in diabetic patients (SMD: -5.83, 95%CI: -15.5, 3.83, p = 0.237, I-square, 97.4%, random effects model, n = 2), but three (SMD: -2.44, 95%CI: -3.37, -1.15, p = 0.001, I-square, 75.9%, random effects model, n = 3) and six months (SMD: -2.41, 95%CI: -3.81, -1.01, p = 0.001, I-square, 78.7%, random effects model, n = 2) after the treatment, a significant decrease is observed in the mean GCF visfatin level. Following the NSPT, the mean visfatin level of GCF, serum and saliva decreases, and in longer follow up time, the level of visfatin decreases significantly. Also, the mean GCF level of diabetic patients decreased after NSPT. Therefore, visfatin level may be used as a diagnostic and therapeutic noninvasive biomarker in patients with periodontitis, which requires further studies.

## Introduction

Periodontal disease is a chronic inflammatory condition that begins with the accumulation of bacterial plaque in the gingival sulcus, which induces an inflammatory response [1–3] and locally, with the destruction of periodontal tissue, it causes bleeding gums, alveolar bone loss, and in some cases causes loss of dental connections [4].

Although microorganisms are considered the main etiological factor in the development of inflammatory lesions, inflammatory mediators also play an essential role in the loss of connective tissue and supporting alveolar bone [5]. Adipose tissue is a highly active endocrine organ that secretes various adipokines and these adipokines participate in a wide range of physiological and pathological processes including inflammation and immunity in the body [6]. One of these adipokines is Visfatin, which has 491 amino acids and was discovered in 2005 and is secreted from adipose tissue, macrophages and leukocytes [7–9].

The presence of visfatin in a wide range of white blood cells and macrophages indicates that visfatin plays an important role in regulating immune function. Visfatin induces the production of pro-inflammatory cytokines [10, 11]. In human bone marrow, as a cytokine-like molecule, it stimulates the early stages of B cell formation [12], it is also secreted by neutrophils in response to pathogens and stimulates monocytes to produce inflammatory mediators [10]. In general, the expression of visfatin increases in inflammatory conditions [13]. In periodontal diseases, as an inflammatory condition, an increase in the amount of visfatin in saliva, serum, gingival crevicular fluid (GCF) and gingival tissue has been reported in various studies [14–20]. According to the results of studies, visfatin can be considered as an inflammatory biomarker in periodontal diseases. [2, 17, 21, 22].

Non-surgical periodontal treatment (NSPT) or phase 1 treatment is the first step in periodontal treatment and its purpose is to change or eliminate microbial etiology and factors involved in gum and periodontal diseases. The common component of all phase 1 treatments includes plaque control, caries control, scaling and root planning (SRP) [23]. SRP is considered as a standard method in NSPT [24], which has been extensively studied to evaluate its effects on periodontal disease and in a review study, it was shown that SRP is effective and reliable [25].

Considering the high prevalence of periodontal disease, it is important to know the pathophysiology and process of the disease in order to diagnose and early treatment. Therefore, investigating the inflammatory process of periodontal disease in relation to visfatin biomarker has been considered and several studies have shown the relationship between visfatin and NSPT. Due to the lack of a systematic review and meta-analysis, in order to reach a consensus on the results of the studies, it was decided to conduct such a study to clarify the diagnostic role of visfatin biomarker in periodontal disease and its relationship with NSPT. The reason for choosing visfatin as a biomarker in periodontitis diseases is that less studies have been done on it, in terms of cost, invasiveness and the method of measurement, it has priority over other inflammatory biomarkers, and in addition, it is a newer biomarker that needs more studies. Therefore, the hypothesis of this study is that the mean visfatin changes before and after NSPT, hence the purpose of this systematic review and meta-analysis is to compare the mean visfatin biomarker level before and after NSPT.

## Methods

### Study design

This study is a systematic review and meta-analysis, which was designed and implemented based on the Cochrane Handbook and reported based on the PRISMA standard checklist.

### Protocol and registration

The protocol of this study has been registered in the international prospective register of systematic reviews (PROSPERO) with ID: CRD42024554967.

### Eligibility criteria

The inclusion criteria included studies that 1) examined the mean and standard deviation of visfatin levels before and after NSPT, 2) periodontal clinical evaluation using the periodontal pocket probe depth (PPD) and clinical attachment loss (CAL) has been investigated [26], 3) clinical trial studies, 4) studies with English full text and 5) published in journals and congresses. The exclusion criteria also include in vitro studies and animal studies, studies with the absence of relevant data and correct data, and lack of access to the full text of the article in English.

### Information sources

To find relevant studies, international databases including PubMed, Web of Science and Scopus were searched according to the search strategy and keywords. Google Scholar was used to review gray literature. Finally, the reference list of the studies included in the meta-analysis was checked manually.

### Searches

The last search was done on June 2024. In order to perform the search, we tried to include relevant keywords. Also, various tools such as tagging, MeSH, and truncation were used in the search in PubMed. The keywords used for the search included "Periodontal Diseases"[Mesh], "Periodontitis"[Mesh], "Chronic Periodontitis"[Mesh], "gingivitis"[Mesh], "periodontal index"[Mesh], "aggressive periodontitis"[Mesh], "Periodontal Diseas*"[tw], "Periodontitis"[tw], "Chronic Periodontitis"[tw], Gingivitis[tw], "Periodontal Index"[tw], "Aggressive Periodontitis"[tw], "periodontal inflammation"[tw], "Therapeutics"[Mesh], "therapy"[Subheading], "non-surgical therapy"[tw], "treat*"[tw], "Pre-B-Cell Colony-Enhancing Factor"[tw], "Therapy"[tw]), Visfatin[tw], "Nicotinamide Phosphoribosyltransferase"[tw], "nicotinamide phosphoribosyltransferase"[Mesh], "NAMPT Protein"[tw], "Colony-Enhancing Factor, Pre-B-Cell"[tw], "Pre B Cell Colony Enhancing Factor"[tw], "NAmPRTase"[tw], "NMN Pyrophosphorylase"[tw], "nicotinamide phosphoribosyltransferase, human" [Supplementary Concept]. The more details of search in three international databases was presented in **Table 1**.

**Table 1:** The search strategy in three databases.

Databases	Query
PubMed	(("Periodontal Diseases"[Mesh] OR "Periodontal Diseas*"[tw] OR "Periodontitis"[Mesh] OR "Periodontitis"[tw] OR "Chronic Periodontitis"[Mesh] OR "Chronic Periodontitis"[tw] OR "gingivitis"[Mesh] OR Gingivitis[tw] OR "periodontal index"[Mesh] OR Periodontal Index[tw] OR "aggressive periodontitis"[Mesh] OR "Aggressive Periodontitis"[tw] OR "periodontal inflammation"[tw]) AND (("non-surgical therapy"[tw] OR "treat*"[tw] OR "Therapy"[tw]) OR ("Therapeutics"[Mesh] AND "therapy" [Subheading]))) AND ("nicotinamide phosphoribosyltransferase"[Mesh] OR "Nicotinamide Phosphoribosyltransferase" [tw] OR Visfatin[tw] OR "NAMPT Protein"[tw] OR "Pre-B-Cell Colony-Enhancing Factor"[tw] OR "Colony-Enhancing Factor, Pre-B-Cell"[tw] OR "Pre B Cell Colony Enhancing Factor"[tw] OR "NAmPRTase"[tw] OR "NMN Pyrophosphorylase"[tw] OR "nicotinamide phosphoribosyltransferase, human" [Supplementary Concept])
Scopus	( (TITLE-ABS-KEY ("Periodontal Diseases") OR TITLE-ABS-KEY ("Periodontitis") OR TITLE-ABS-KEY ("Chronic Periodontitis") OR TITLE-ABS-KEY ("Periodontal Disease") OR TITLE-ABS-KEY ("gingivitis") OR TITLE-ABS-KEY ("periodontal index") OR TITLE-ABS-KEY ("Aggressive Periodontitis") OR TITLE-ABS-KEY ("periodontal inflammation") ) ) AND ( (TITLE-ABS-KEY ("non-surgical therapy") OR TITLE-ABS-KEY ("treatment") OR TITLE-ABS-KEY ("Therapy") OR TITLE-ABS-KEY ("Therapeutics") OR TITLE-ABS-KEY ("non surgical therapy") OR TITLE-ABS-KEY ("non surgical periodontal therapy") ) ) AND ( (TITLE-ABS-KEY ("nicotinamide phosphoribosyltransferase") OR TITLE-ABS-KEY (visfatin) OR TITLE-ABS-KEY ("NAMPT Protein") OR TITLE-ABS-KEY ("Pre-B-Cell Colony-Enhancing Factor") OR TITLE-ABS-KEY ("Colony-Enhancing Factor, Pre-B-Cell") OR TITLE-ABS-KEY ("Pre B Cell Colony Enhancing Factor") OR TITLE-ABS-KEY ("NAmPRTase") OR TITLE-ABS-KEY ("NMN Pyrophosphorylase") ) )
Web of sciences	"nicotinamide phosphoribosyltransferase" (Topic) or "Nicotinamide Phosphoribosyltransferase" (Topic) or Visfatin (Topic) or "NAMPT Protein" (Topic) or "Pre-B-Cell Colony-Enhancing Factor" (All Fields) or "Colony-Enhancing Factor, Pre-B-Cell" (All Fields) or "Pre B Cell Colony Enhancing Factor" (All Fields) or "NAmPRTase" (All Fields) or "NMN Pyrophosphorylase" (All Fields) AND "non-surgical therapy" (Topic) or "treatment" (Topic) or "Therapy" (Topic) or "Therapeutics" (Topic) or "non surgical therapy" (Topic) or "non surgical periodontal therapy" (Topic) AND TS = ("Periodontal Diseases") OR TS = ("Periodontitis") OR TS = ("Chronic Periodontitis") OR TS = ("Periodontal Disease") OR TS = ("gingivitis") OR TS = ("periodontal index") OR TS = ("Aggressive Periodontitis") OR TS = ("periodontal inflammation")

### Study selection

The articles retrieved in the search phase were first entered into the Endnote software, and duplicate articles were identified and removed using this software. According to the Cochrane protocol, in the next step, the title and abstract of the articles were screened, and unrelated articles were removed, and the full text of related or suspicious articles were evaluated in the next stage, and finally, the required data from the articles that met the criteria for entering the meta-analysis had been extracted. These steps were carried out by two authors, and in case of disagreement between them, a consensus was reached with the guidance of the supervisor.

### Data collection process and data items

The data were extracted by two authors, and in case of disagreement, a consensus was reached by the supervisor. The authors of the articles whose full text was not available or the reported information had a problem were contacted. From the articles that met the inclusion criteria, the first author, the year of publication of the article, the country, the number of people included in the study, the mean age of the participants, the method of measuring visfatin level, the methodological quality score of the articles and the main conclusions including the mean and standard deviation (SD) of the visfatin level before and after NSPT was extracted.

### Risk of bias in individual studies

To check the risk of methodological bias, the NIH quality assessment tool [27] was used, which is specially designed for before and after studies without an external control group. In this checklist, the quality of studies is evaluated using 12 items. For each of these 12 items, one of the options "yes", "no" or "cannot be determined (CD) / not reported (NR) / not applicable (NA)" is selected, and finally the articles are categorized as good, fair and poor. A score less than 5 was classified as poor, between 5 and 8 as fair, and above 8 was classified as good. For more details, these 12 items included questions about these items: study question, eligibility criteria and study population, study participants, eligible participants, sample size, intervention, outcome, blinding, follow-up rate, statistical analysis, multiple outcome measures and group-level interventions and individual-level outcome efforts.

### Summary measures and synthesis of results

Considering that the aim of this study is to compare the mean visfatin level before and after NSPT, the desired effect size is standardized mean difference (SMD) along with 95% confidence interval (95%CI), which are calculated as the summary measures. To perform the meta-analysis, it was first checked whether there is significant heterogeneity between the articles and in cases where there was significant heterogeneity between the studies, the random-effects model was used, otherwise the fixed-effects model was used. To check the heterogeneity between the studies, the I-square statistic and the Chi-square test were used, and an I-square above 0.7 was considered as significant heterogeneity. In cases where the value of the I-square was higher than 0.7, the random-effects model was used, and in the cases where the value of the I-square was less than 0.7, the fixed-effects model was used for data synthesis.

### Additional analyses

As additional analyses, considering that the duration of follow-up after NSPT was different in the studies, analyzes were performed based on the duration of follow-up separately. Visfatin levels were also compared before and after treatment for GCF, serum and saliva. In addition, in 5 articles, there were studies on people with periodontitis and type 2 diabetes (T2DM), and these 5 studies were meta-analyzed separately.

## Results

### Study selection

**Fig 1:**
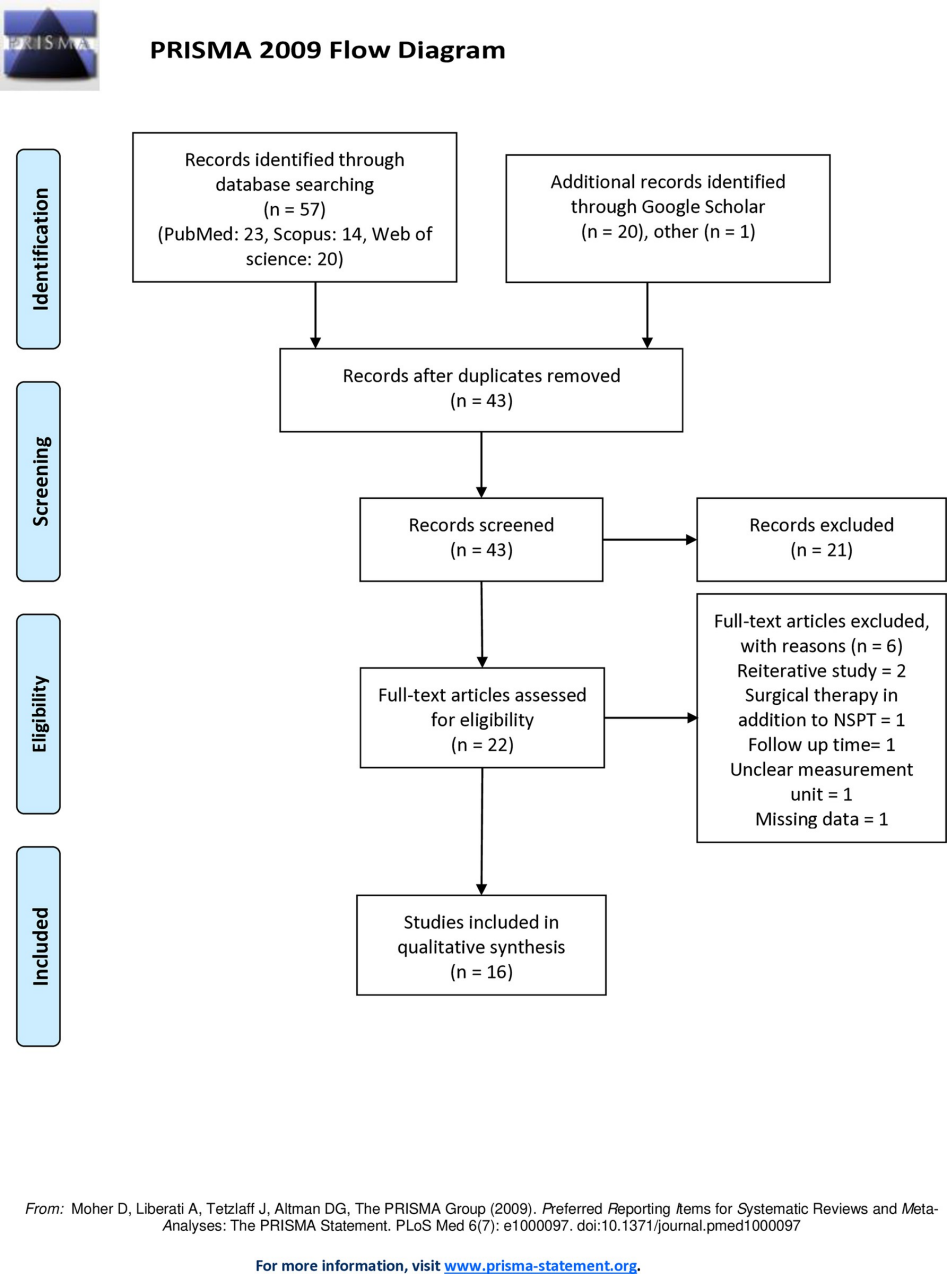
Flow chart of the literature search for studies included in meta-analysis.

The flowchart of screening and selection of studies is shown in **Fig 1**. 57 studies were retrieved from the databases search and 21 studies were retrieved from the gray literature (78 studies in total). After removing duplicate studies, the title and abstract of the remaining 43 studies were screened and 21 studies were excluded. In the next step, the full text of the remaining 22 studies was evaluated, and 6 studies were excluded due to lack of inclusion criteria, and 16 studies [18, 22, 28–41] were included in the meta-analysis. The excluded studies mostly did not have the required data, had performed surgical treatments, and the study design did not match our objectives, or did not meet other inclusion criteria. More details about the removed articles are specified in the **S1 Appendix**.

### Study characteristics

The details of the characteristics of the included articles are reported in **Table 2** and also, more details about the articles included in the meta-analysis are given in the **S2 Appendix**. The oldest article was published in 2012 and the most recent in 2023. Most of the studies were conducted in India and the sample size varied from 9 to 30. In all studies, ELISA kit was used to measure visfatin, and NSPT treatment was SRP and oral hygiene instructions (OHI) in the majority of studies.

**Table 2:** A summary of the included study characteristics.

Author	Year	Country	Group	Sample Size	NSPT	Meanage	Quality score	GCF sampling	Saliva sampling	Serum sampling	ELISA kit
Raghavendraa N.M [18]	2012	India	Periodontitis	15	SRP	39	7	White color-coded 1–5 μl calibrated volumetricmicrocapillary pipettes	_	Two milliliters of blood was collected from the antecubital fossa by venipuncture using a 20-gauge needle with 2 ml syringe	RayBio, Parkway Lane, GA, USA
Wu Y [22]	2015	China	Periodontitis & T2DM	23	SRP, OHI	54	7	Paper strips (Whatman Paper Ltd., England)	_	2ml of blood was drawn before breakfast on the day of GCF sampling	EIA-VIS-1, RayBio, RayBiotech, Norcross, USA
Abolfazli N [28]	2015	Iran	Periodontitis	18	SRP, OHI	41	7	_	Passive drooling method was used to collect un-stimulated whole saliva samples	Blood samples were taken from the forearm veins using 5-ml syringes	Glory Science Co., Ltd, USA
Tabari Z.A [40]	2015	Iran	Periodontitis	20	SRP, OHI	38	6	_	5 ml of unstimulated whole expectorated saliva was collected from each participant, according to a modification of the method described by Navazesh	_	Human Visfatin (VISFATIN) ELISA Kit (Cat. No. E0025Hu)
Mishra V [33]	2016	India	Periodontitis	14	SRP, OHI	42	6	Microcapillary pipette (Top-Tech biomedicals, Mumbai)	_	_	Genexbio Company, New Delhi, India
Ozcan E [35]	2016	Turkey	Periodontitis	17	SRP, OHI	42	6	_	Approximately 5 ml of saliva was collected according to the unstimulated saliva collection procedure	_	Not mentioned
Mishra V [33]	2016	India	Periodontitis & T2DM	14	SRP, OHI	48	6	Microcapillary pipette (Top-Tech biomedicals, Mumbai)	_	_	Genexbio Company, New Delhi, India
El-Sharkawy H.M [30]	2017	Egypt	Periodontitis	17	SRP, restoration of carious lesions and food impaction areas, extraction of hopeless teeth, OHI	48	7	_	Whole unstimulated saliva samples (5 ml) were collected by asking the patients to expectorate into polypropylene tubes	_	R&D systems, Minneapolis, USA
Cetiner D [29]	2018	Turkey	Periodontitis	9	Phase I therapy, OHI	44	7	Paper strips (Periopaper, ProFlow Inc., Amityville, NY, USA)	_	_	ELx800, Biotek, Winooski, VT, USA
Nilamahan A [34]	2019	India	Periodontitis	18	SRP, OHI	46	7	_	Unstimulated whole expectorated saliva (5 ml) was collected according to the drooling method standardized by Navazesh	_	Not mentioned
Dalia M [31]	2019	Egypt	Periodontitis	30	SRP, OHI	30	7	Paper strips	_	Not mentioned	EIAab (China) catalog no.: E0638h
Dalia M [31]	2019	Egypt	Periodontitis & T2DM	30	SRP, OHI	30	7	Paper strips	_	Not mentioned	EIAab (China) catalog no.: E0638h
El Makaky Y.M [38]	2020	Egypt	Periodontitis & T2DM	15	SRP, OHI		8	Paper strips (Whatman Paper, Little Chalfont, UK)	_	_	Visfatin Enzyme Immunoassay, Ray Biotech, Norcross, GA
Ziaei N [41]	2020	Iran	Periodontitis & T2DM	20	SRP, OHI	52	8	_	5 mL of unstimulated whole saliva was collected by spitting method	_	Biovendor Research & Diagnostic Products: USA
El Makaky Y.M [38]	2020	Egypt	Periodontitis	15	SRP, OHI		8	Paper strips (Whatman Paper, Little Chalfont, UK)	_	_	Visfatin Enzyme Immunoassay, Ray Biotech, Norcross, GA
Saseendran G [37]	2021	India	Periodontitis	16	SRP		5	_	About 2 ml of unstimulated saliva was collected from each participant	_	Human Visfatin ELISA Kit, Bioassay Technology Laboratory
Rajasekar A [36]	2023	India	Periodontitis	20	SRP, OHI	41	7	_	5 mL of unstimulated whole saliva collected using spitting method	_	Elabscience Human VF ELISA kit, United States of America
Surya D [39]	2023	India	Periodontitis	30	SRP, OHI	45	7	_	4 ml of whole unstimulated saliva sample was obtained using the modified draining method	4 mL of blood was obtained by venepuncture from the antecubital region	Elabscience, Houston, Texas, United States of America
Mallick S [32]	2023	India	Periodontitis	20	SRP, OHI	41	9	_	10 ml of unstimulated saliva was collected using the drainage method as suggested by Navazesh.	_	Raybio visfatin EIA KIT Cat.no—EIA-VIS/EIAM-IS/EIAR-VIS

### Risk of bias within studies

Risk of bias within studies was checked using the NIH tool, the results of which are shown in detail in **Table 1**. Based on this checklist, one study had good quality and 15 studies had fair quality. More details about the items and the quality score of the included studies are specified in **S3 Appendix**.

### Results of individual studies and synthesis of results

The results of the meta-analysis and heterogeneity are shown in **Table 3**, separating the type of sample and the duration of follow-up. In general, in all samples and in all follow-up times, after NSPT, the mean level of visfatin decreased, which was significant in most of the follow-up times and samples. The results show that one month after the NSPT, there was no significant difference in the mean visfatin level of GCF (SMD: -3.91, 95%CI: -9.83, 2.01, p = 0.195, I-square, 96.6%) and serum (SMD: -0.33, 95%CI: -0.98, 0.33, p = 0.332), but 2, 3 and 6 months after NSPT, the mean visfatin level of GCF and serum decreased significantly.

**Table 3:** Summary of meta-analysis results to compare visfatin level before and after NSPT among cases with periodontitis.

Sample	Follow up time	Meta-analysis				Heterogeneity		
		No of studies	SMD (95%CI)	P value	Model	Chi square	P value	I-square (%)
GCF	One month	2	-3.91 (-9.83, 2.01)	0.195	Random	29.45	0.001	96.6%
	Two months	1	-9.68 (-12.3, -7.05)	0.001	Fixed	-	-	-
	Three months	3	-1.72 (-2.77, -0.68)	0.001	Random	9.4	0.009	78.7%
	Six months	1	-3.60 (-4.78, -2.42)	0.001	Fixed	-	-	-
Serum	One month	1	-0.33 (-0.98, 0.33)	0.332	Fixed	-	-	-
	Two months	2	-6.38 (-10.4, -2.34)	0.002	Random	10.11	0.001	90.1%
	Three months	1	-3.38 (-4.18, -2.59)	0.001	Fixed	-	-	-
	Six months	0	-	-	-	-	-	-
Saliva	One month	2	-0.65 (-1.11, -0.19)	0.006	Fixed	0.42	0.515	0.0%
	Two months	3	-5.68 (-12.9, 1.59)	0.126	Random	158.5	0.001	98.7%
	Three months	4	-3.25 (-5.45, -1.05)	0.004	Random	72.6	0.001	95.9%
	Six months	1	-0.80 (-1.5, -0.10)	0.025	Fixed	-	-	-

**Fig 2:**
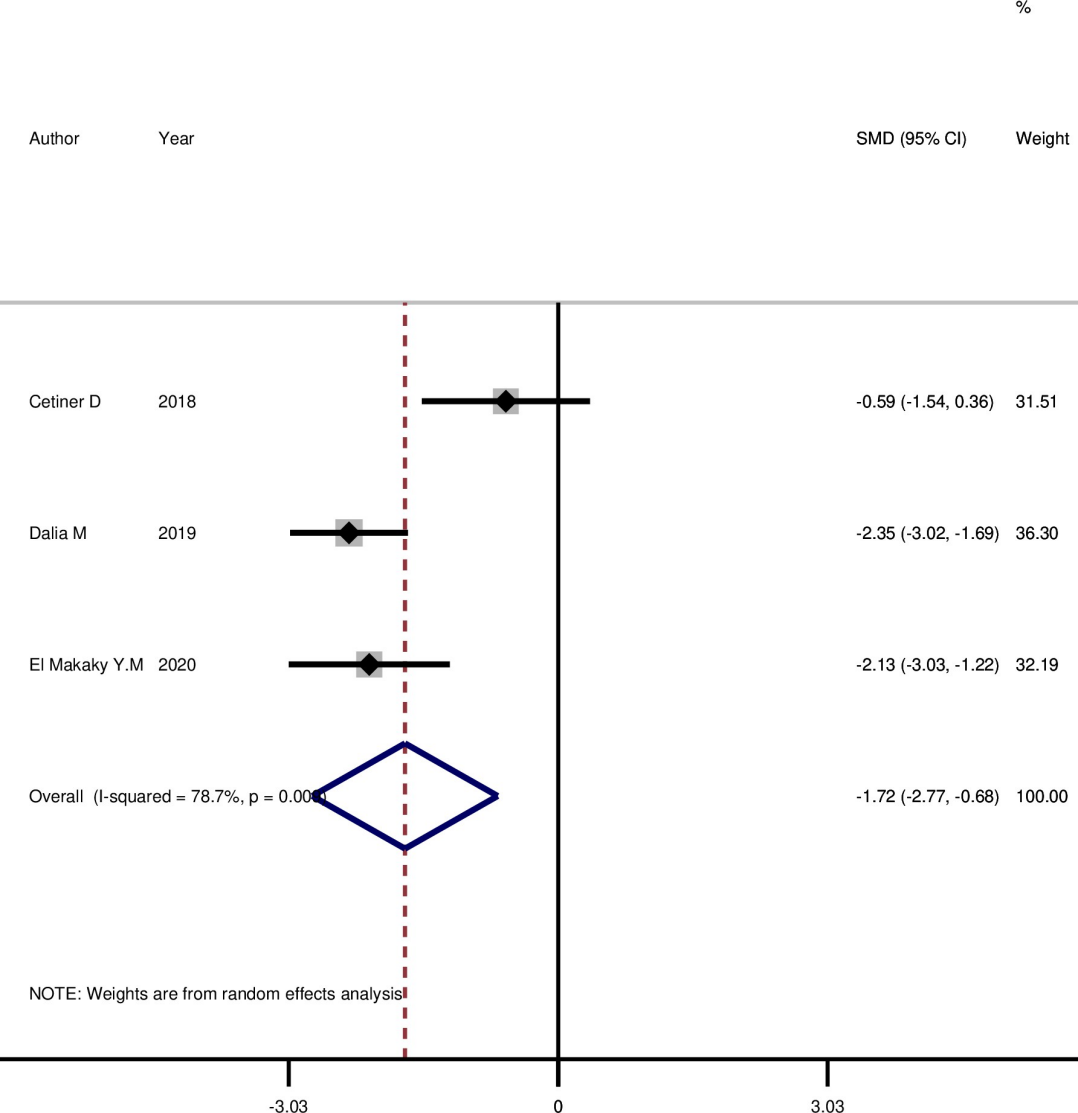
The comparison of GCF visfatin level before and three months after NSPT among cases with periodontitis.

**Fig 3:**
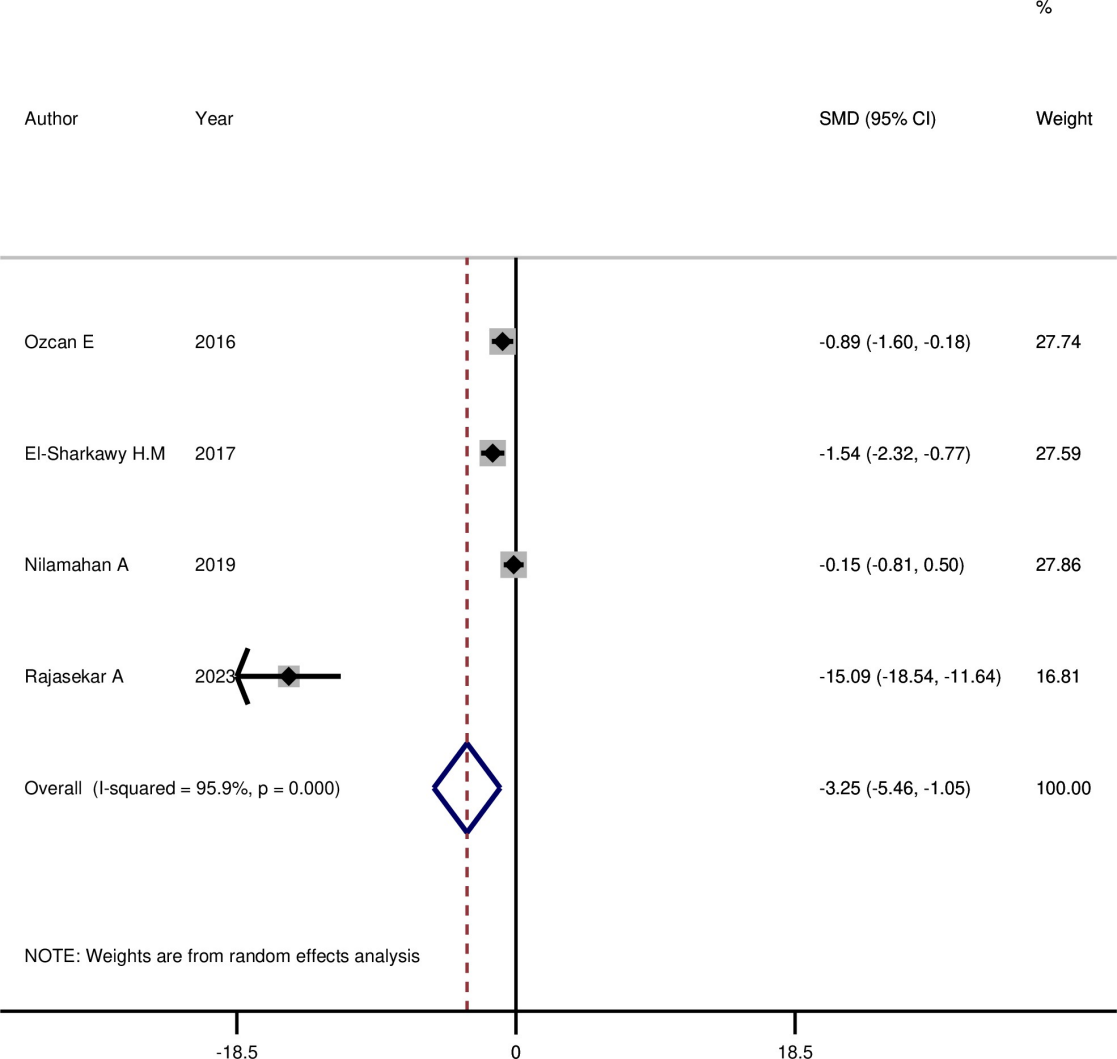
The comparison of saliva visfatin level before and three months after NSPT among cases with periodontitis.

As shown in **Fig 2**, the mean visfatin level of GCF decreased significantly 3 months after NSPT (SMD: -1.72, 95%CI: -2.77, -0.68, p = 0.001), and the mean saliva visfatin level **(Fig 3)** also showed a significant decrease 3 months after NSPT (SMD: -3.25, 95%CI: -5.45, -1.05, p = 0.004).

### Additional analysis

**Fig 4:**
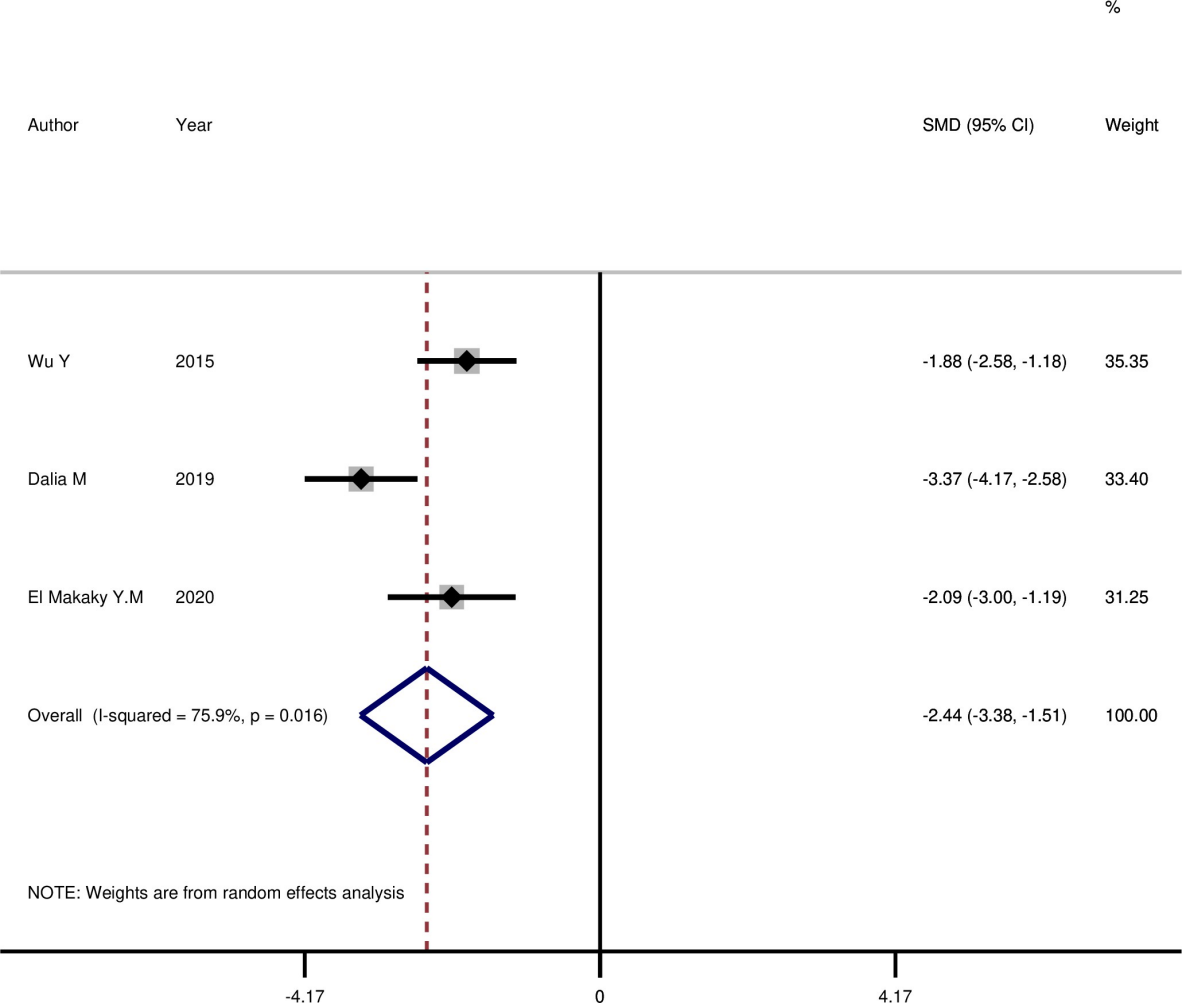
The comparison of GCF visfatin level before and three months after NSPT among cases with periodontitis and T2DM.

In **Table 4**, the comparison of Visfatin GCF level before and after NSPT treatment in T2DM patients is reported. Similar to the previous results, there is no significant difference one month after NSPT in diabetic patients (SMD: -5.83, 95%CI: -15.5, 3.83, p = 0.237, I-square, 97.4%), but three (SMD: -2.44, 95%CI: -3.37, -1.15, p = 0.001, I-square, 75.9%) (**Fig 4**) and six months (SMD: -2.41, 95%CI: -3.81, -1.01, p = 0.001, I-square, 78.7%) after the treatment, a significant decrease is observed in the mean visfatin GCF level.

**Table 4:** Summary of meta-analysis results to compare visfatin level before and after NSPT among cases with periodontitis and T2DM.

Sample	Follow up time	Meta-analysis				Heterogeneity		
		No of studies	SMD (95%CI)	P value	Model	Chi square	P value	I-square (%)
GCF	One month	2	-5.83 (-15.5, 3.83)	0.237	Random	37.84	0.001	97.4%
	Three months	3	-2.44 (-3.37, -1.51)	0.001	Random	8.29	0.016	75.9%
	Six months	2	-2.41 (-3.81, -1.01)	0.001	Random	4.70	0.030	78.7%

Sensitivity analysis was also performed for the average changes in salivary visfatin levels 3 months after NSPT treatment, and the results showed that the biggest change in the results was observed after the withdrawal of Rajasekar A et al.’s study, although the changes were still significant (SMD: -0.84, 95%CI: -1.62, -0.05).

## Discussion

The results of this study showed that following the NSPT, the mean level of visfatin decreases, and the more time passes after the treatment, the level of visfatin decreases significantly. A decrease was observed in the mean visfatin level of GCF, serum and saliva. Also, in a separate analysis, similar results were obtained for diabetics, so that the mean GCF level of diabetic patients significantly decreased after NSPT. Therefore, visfatin level of GCF, serum and saliva may be used as a diagnostic and therapeutic noninvasive biomarker in patients with periodontitis.

Regarding the clinical importance of visfatin measurement as a diagnostic and therapeutic biomarker, it can be mentioned that compared to other diagnostic methods, the sampling and measurement to assess the visfatin is less invasive and less expensive and can be more accessible than clinical methods. Also, visfatin measurement can be done by a laboratory technician, but the diagnosis of periodontitis is clinically and more specialized by an experienced dentist.

Periodontitis is an inflammatory disease and involves a severe inflammation of the periodontium [42–44], and available evidence suggests that topical treatment of periodontitis improves other markers of comorbid conditions [44]. On the other hand, visfatin is known as one of the inflammatory markers and can probably preserve inflammation in immune cells [45], therefore, in this study, our hypothesis was that the level of visfatin can change before and after the NSPT and the obtained results showed evidence in favor of our hypothesis. In addition to being secreted from fat tissue, visfatin is also secreted from other places, especially inflammatory cells. Therefore, visfatin has a wide range of local and systemic effects, one of which is inflammation [46], so it can play a role in inflammatory diseases such as periodontitis. In another study, Xiao et al. [47] has shown that more severe periodontal disease is associated with higher levels of visfatin in GCF and gingival tissues, therefore, visfatin has been proposed as a biomarker for periodontitis. Also, the study of Bengi et al. has shown that visfatin can play a role in periodontal disease [48].

In Zhu et al.’s study [49], it has been reported that visfatin levels in GCF, serum and saliva increase following periodontitis, which probably means that visfatin levels in GCF, serum and saliva can decrease after periodontal treatment. Also, by conducting a meta-analysis study, Bayani M et al [2] concluded that the visfatin increases in periodontal diseases and has an effective role in the inflammatory process of these diseases and visfatin level of GCF, serum and saliva can be used as a diagnostic biomarker of periodontitis.

The role of visfatin in other diseases has also been studied. In a meta-analysis study by Jiang Y et al. [50], the results have shown that visfatin levels in patients with MS are significantly higher than healthy subjects in the control group, and maybe visfatin can be used to predict the occurrence of MS. Also, the results of Mohammadi et al.’s study showed that visfatin expression is associated with poor clinical outcomes in tumor patients, so that high visfatin expression can act as a probable biomarker for metastasis and poor prognosis in different types of cancers [51]. In addition, in the study of Zou et al., it has been shown that visfatin serum level is significantly higher in psoriasis patients compared to healthy individuals, and a positive correlation was also observed between the severity of psoriasis and visfatin serum level [52].

In 2019 in India, Mopidevi A et al investigated salivary visfatin level in patients with chronic periodontitis before and after periodontal treatment. Periodontal characteristics including plaque index, gingival index, sulcus bleeding index, probe depth and clinical adhesion level were recorded at the beginning. The level of salivary visfatin was evaluated before and after the 12-week period of periodontal treatment. The results showed that salivary visfatin significantly decreased after periodontal treatment, so the level of salivary visfatin can be used as a marker to evaluate the response to periodontal treatment [53]. In another study, Türer Ç et al [54] in 2016 in Turkey investigated the effect of non-surgical periodontal treatment on visfatin levels in GCF and serum of periodontal patients. Gingival crevice fluid and serum samples were collected before treatment and in the first, third and sixth months after treatment, and finally the results showed that non-surgical periodontal treatment leads to a significant reduction in visfatin serum and gingival crevice fluid levels. Visfatin level increased with periodontal disease and decreased with its treatment.

One of the limitations of this study was data heterogeneity. Among the reasons for heterogeneity, we can mention different methods of saliva sampling and measurement, which probably different tools can affect the results. Also, reporting the amount of visfatin with a standard unit (for example, picograms per milliliter) can also help reduce heterogeneity. We included a total of 16 related articles in the analysis, but because the analyzes were performed separately at different follow-up times (1, 2, 3 and 6 months), and also the analyzes were performed based on the type of sample (serum, saliva, and GCF), in some analyzes the number of articles was not enough, therefore, conducting more studies are recommended to generate rigorous causal evidence. Another limitation of this study was that the articles included in the meta-analysis were limited to a few countries and there were fewer studies from developed countries. There is a possibility that visfatin levels and its relationship with periodontitis are different in different ethnicities and races due to the interaction of possible genes. Therefore, it is recommended to conduct studies in different countries to achieve more valid results. Finally, in this study, we searched 3 international databases and to find more relevant articles, it is recommended to search more databases in future studies.

## Conclusion

Following the NSPT, the mean visfatin level of GCF, serum and saliva decreases, and the more time passes after the treatment, the level of visfatin decreases significantly. Also, in a separate analysis, similar results were obtained for diabetics, so that the mean GCF level of diabetic patients significantly decreased after NSPT. Therefore, visfatin level may be used as a diagnostic and therapeutic noninvasive biomarker in patients with periodontitis, which requires further studies.

## Supporting information

S1 Checklist PRISMA 2009 checklist. (DOC)

S1 Appendix List of excluded studies. (XLSX)

S2 Appendix List of Included studies. (XLS)

S3 Appendix NIH score. (XLSX)
